# Identifying individual and organizational predictors of accidental exposure to blood (AEB) among hospital healthcare workers: A longitudinal study

**DOI:** 10.1017/ice.2023.248

**Published:** 2024-04

**Authors:** René Sosata Bun, Karim Aït Bouziad, Oumou Salama Daouda, Katiuska Miliani, Anastasia Eworo, Florence Espinasse, Delphine Seytre, Anne Casetta, Simone Nérome, Laura Temime, Mounia N. Hocine, Pascal Astagneau

**Affiliations:** 1 IPLESP, INSERM, Sorbonne University, Paris, France; 2 INSERM CIC1410, CHU Réunion, Saint-Pierre, France; 3 MESuRS Laboratory, Conservatoire National des Arts et Métiers, Paris, France; 4 CPIAS Ile de France, Paris, France; 5 Hôpital Ambroise Paré, Boulogne, France; 6 Hôpital Avicenne, Bobigny, France; 7 Hôpital Cochin, Paris, France; 8 Hôpital Beaujon, Clichy, France; 9 PACRI Unit, Institut Pasteur, Conservatoire National des Arts et Métiers, Paris, France

## Abstract

**Background:**

Accidental exposure to blood (AEB) poses a risk of bloodborne infections for healthcare workers (HCWs) during hospital activities. In this study, we identified individual behavioral and organizational predictors of AEB among HCWs.

**Methods:**

The study was a prospective, 1-year follow-up cohort study conducted in university hospitals in Paris, France. Data were collected from the Stress at Work and Infectious Risk in Patients and Caregivers (STRIPPS) study. Eligible participants included nurses, nursing assistants, midwives, and physicians from 32 randomly selected wards in 4 hospitals. AEB occurrences were reported at baseline, 4 months, 8 months, and 12 months, and descriptive statistical and multilevel risk-factor analyses were performed.

**Results:**

The study included 730 HCWs from 32 wards, predominantly nurses (52.6%), nursing assistants (41.1%), physicians (4.8%), and midwives (1.5%). The incidence rate of AEB remained stable across the 4 visits. The multilevel longitudinal analysis identified several significant predictors of AEB occurrence. Individual-level predictors included younger age, occupation as nurses or midwives, irregular work schedule, rotating shifts, and lack of support from supervisors. The use of external nurses was the most significant ward-level predictor associated with AEB occurrence.

**Conclusions:**

AEBs among HCWs are strongly associated with organizational predictors, highlighting the importance of complementing infection control policies with improved staff management and targeted training. This approach can help reduce AEB occurrences and enhance workplace safety for HCWs.

Accidental exposure to blood (AEB) poses a potential risk for bloodborne pathogen infections in healthcare workers (HCWs) and subsequently in patients during care. The most common pathogens transmitted through AEB include human immunodeficiency virus (HIV), hepatitis B and C, and certain bacteria.^1^ AEBs can occur in various ways, such as percutaneous needlestick injuries, cuts, scrapes, or splashes to the mucous membranes like the eyes, nose, or mouth. Needlestick injuries are the most common cause of AEB during care tasks.^2^ High-risk care tasks for AEBs typically involve handling sharp objects, administering medications, managing bodily fluids, and working with contaminated equipment. However, AEB can also occur during other nursing tasks and environmental activities, such as cleaning contaminated surfaces or disposing of hazardous waste. To mitigate AEBs, it is vital to adhere to standard precautions and to utilize personal protective equipment, including gloves, masks, and eye protection, during high-risk care. Proper disposal of needles and other sharp objects is also essential.^3^ AEB serves as a marker for risk, quality, and safety of care among HCWs because the accident rate might reflect the performance of healthcare facilities in terms of cost and economic impact.^4^ AEB risk increases during nursing care or environmental activities, particularly for HCWs regularly exposed to invasive procedures with significant blood contact.^5^ Activities like wound care, equipment handling, and waste disposal can raise the likelihood of AEB, which contributes to stress and has physical and psychological consequences for HCWs. However, understanding the impact of various healthcare-related factors on AEB risk remains challenging. Although behavioral and management factors influence the risk of healthcare-associated infections in patients,^6^ research identifying these factors for HCWs has been limited. Studies highlight the importance of teamwork, leadership, and proper equipment,^7^ as well as the need for a strong safety culture among HCWs.^8^ Considering this evidence, a French cross-sectional study^9^ examined the impact of various factors on HCW stress and fatigue in intensive care units (ICUs). These researchers found that employment and organizational factors were significantly associated with stress and fatigue outcomes, even after controlling for demographic factors. Addressing factors at both individual and organizational levels is crucial for improving HCWs health. With this background in mind, we sought to identify both individual behavioral and organizational factors that could influence the occurrence of AEB in HCWs and to better elucidate the role of various potential risk factors.

## Methods

### Study design and participants

The STRIPPS study (no. NCT03532321) was a 1-year follow-up multicenter, prospective study investigating the individual and organizational factors that predict occupational exposure to blood among 730 HCWs in Paris university hospitals.^10^ The study was carried out in 4 general-care hospitals between February 2018 and July 2019 and included nurses, nursing assistants, midwives, and physicians as participants. We included both permanent and fixed-term contract HCWs with work contracts lasting at least 1 year, matching the survey duration. We excluded those with contracts <1 year, as well as external personnel (eg, nursing float pool). The sample size was a convenience sample based on a previous study.^9^ Data were collected from these HCWs by randomly selecting 8 wards per participating hospital from those that employed at least 30 HCWs.

### Data collection

Data were collected in all participating individuals, every 4 months for a total follow-up period of 1 year, by 2 different interviewers. The collection times were designated as t0, t1, t2, and t3, corresponding to the first collection during the inclusion visit and follow-up visits at 4, 8, and 12 months, respectively. For the first collection (t0), dates and times of visits were randomly assigned for each ward. For subsequent collections, individual appointments were made considering different work shifts (day and night) to ensure a comprehensive representation of HCW schedules.

Data were collected through questionnaire-based interviews at both the ward and individual levels. Potential participants were informed of the study through an information letter and gave verbal consent at the beginning of each interview. Participants were guaranteed confidentiality and anonymity of responses.

### Ward-level variables

The hospital health executives (nurse managers) were interviewed at t0 to collect data for each of the 32 wards. The data collected pertained to the medical specialty of the ward, the number of beds per ward, the proportion of double rooms, the frequency of tasks performed outside the ward, the ratio of HCW to patients, and the use of external staff. Further inclusion and exclusion criteria were previously reported.^11^

### Individual-level variables

Interviewers collected a range of information about the HCWs including demographics and details about their work organization. Validated questionnaires, such as the Effort-Reward Imbalance (ERI),^12^ the Job Content Questionnaire (JCQ),^13^ the Perceived Stress Scale-10 (PSS-10),^14,15^ and the Pichot fatigue scale,^16^ were used to standardize measures of overcommitment, social support, and stress and fatigue levels, respectively. This information was collected at 4 different times (ie, t0, t1, t2, and t3) to track changes over time.

### Outcome

The outcome variable was the self-reported occupational exposure to blood among HCWs. An AEB is defined as any unintended contact with blood or blood-containing body fluids, which can occur through percutaneous injuries, cuts, scrapes, or splashes to the skin or mucous membranes. At each visit, HCWs were asked about the number of AEBs they had experienced within the previous 4 months. Only declared AEBs were considered. The accidents were further described in terms of their context, cause, and nature of injury. Notably, this information was obtained through self-reporting by the HCWs during the study visits.

### Missing data

To handle missing data, we utilized multiple imputation on validated questionnaire items (JCQ, PSS-10, Pichot, and ERI questionnaires) with the R mice package.^17^ The imputation was performed on both continuous and categorical variables in longitudinal data. Missing data for all questionnaire items in the imputation model were assumed to be missing at random.

### Statistical analyses

First, we conducted a descriptive analysis to summarize the data collected at the individual and ward levels. We assessed changes in individual-level variables over time using 2-sided student tests for continuous variables and χ^2^ tests for categorical variables. Next, we identified factors associated with AEBs in participating HCWs. Bivariate analyses were conducted on all individual-level variables to determine which variables were relevant for inclusion in the multivariate analysis. Variables with *P* ≤ .20 were considered for inclusion in the model. Based on these results, we performed a multivariate analysis with longitudinal data to investigate the association between risk factors and the outcome variable. We used a linear mixed-effects model, including the hospital as a random effect to account for the unobserved heterogeneity across hospitals. We used the hospital variable as the random effect because it represents a higher-level grouping in the data hierarchy and demonstrated a significant effect in the bivariate analysis. To select the best model, we used the Akaike information criterion (AIC) and compared it with alternative models to ensure the inclusion of the most suitable random and fixed effects. All data analyses were conducted using R package *lme4* software (R Foundation for Statistical Computing, Vienna, Austria).^18^

### Ethical approval

The study protocol obtained both an agreement from the French Committee for the Protection of Persons (CPP) on November 14, 2017, and clearance from the French Data Protection Authority (CNIL) on December 14, 2017 (IDRCB no. 2017-A02939-44).

## Results

### Demographic and work characteristics of the study sample

In this study, a sample of 730 HCWs was analyzed. The sample comprised 384 nurses (52.6%), 300 nursing assistants (41.1%), 35 physicians (4.8%), and 11 midwives (1.5%). The female:male sex ratio was 5:1, with 610 female respondents (83.6%). The majority of HCWs were permanent staff (n = 644, 88.2%) compared to temporary staff (n = 66, 9.0%) and contractual staff (n = 19, 2.6%). The average number of years of experience was 10.5 (±9.7), and 380 respondents (52.1%) had supervising responsibilities. On average, HCWs worked 37.6 hours per week (±5.8 hours), and 614 (84.1%) had advance knowledge of their schedule, but 322 (44.1%) had never participated in creating it. Furthermore, 302 (41.4%) staff did not take their rest immediately after night shifts. In terms of transportation, 365 participants (50.0%) reported a daily car use versus 303 (41.5%) using public transportation and 62 (8.5%) using other options (ie, walking, biking, or motor biking). The daily commute duration was <1 hour for 306 participants (41.9%), between 1 and 2 hours for 321 participants (44.0%), and >2 hours for 103 participants (14.1%).

### Characteristics of participating wards in the study

This study included 32 wards from various medical fields, including surgery and obstetrics (14 wards, 43.8%), medicine (11 wards, 34.4%), and ICUs (7 wards, 21.9%). The average number of beds per ward was 35.5 (±18.5), and ∼20% of ward rooms were double rooms. The patient-to-physician ratio and patient-to-paramedic ratio were 2.9 and 0.8, respectively. The scheduling of work varied across participating wards. Most HCWs (80%) organized work in three 8-hour shifts, whereas 16% of wards used two 12-hour shifts. More than 80% of wards required HCWs to leave the ward on occasion, and most wards utilized interim staff.

### Details of accidental exposures to blood

In total, 108 instances of occupational blood exposure were reported among 71 HCWs. Table 1 provides details about AEBs, including the nature of the injury, the mechanism of occurrence, and the task being performed when the accident occurred, grouped by medical specialty and occupation. Of the 108 reported blood accidents, 52 occurred among 29 HCWs in the ICU, 40 among 37 HCWs in surgery and obstetrics, and the remaining 16 among 5 HCWs in other medical specialties. The incidents included 59 splashes, 44 needlestick injuries, and 5 cuts from sharp objects. The main reasons were handling a mounted needle (57%), followed by handling contaminated instruments (17%) and other mechanisms (13%). Most injuries occurred during tasks such as blood sampling (41%), infusion (12%), surgery (12%), nursing and hygiene (11%), and other care (9%). Surgeons and midwives, who carry out procedures involving skin punctures or cuts in surgery and obstetrics departments, had higher rates of occupational blood exposure, at 37.5% and 20%, respectively. ICU nurses had the second-highest rate of blood exposure, at 10.8%.

**Table 1.** Incidence Rate of Accidental Exposure to Blood per 1,000 Person Years by Medical Specialty and by Occupation (n=108)

Characteristics of occupational	By Medical Specialty			By Occupation			Total^a^
Exposures to blood	Intensive Care Unit (n=203)^a^	Surgery Obstetrics (n=314)^a^	Nurses (n=384)^a^	Nursing Assistants (n=300)^a^	Physicians (n=35)^a^	Midwives (n=11)^a^	
No. of visits	1,128	755	1070	1365	138	40	2,613
**Nature of injury**							
Splash	38.4 (28)	20.4 (23)	35.9 (49)	4.7 (5)	36.2 (5)	…	22.6 (59)
Needlestick injury	30.1 (22)	12.4 (14)	20.5 (28)	4.7 (5)	29 (4)	175 (7)	16.8 (44)
Cut by other sharp object	2.7 (2)	2.7 (3)	1.5 (2)	1.9 (2)	…	25 (1)	1.9 (5)
**Mechanism of accident occurrence**							
By handling a mounted needle	35.6 (26)	22.2 (25)	35.9 (49)	1.9 (2)	29 (4)	175 (7)	23.7 (62)
By handling soiled instruments	21.9 (16)	0.9 (1)	10.3 (14)	3.7 (4)	…	…	6.9 (18)
By handling a mounted or unmounted syringe	4.1 (3)	1.8 (2)	4.4 (6)	…	…	…	2.3 (6)
By handling samples	5.5 (4)	0.9 (1)	2.9 (4)	…	…	25 (1)	1.9 (5)
By intervening on a device	…	…	1.5 (2)	0.9 (1)	…	…	1.1 (3)
Other mechanisms at the origin of contact with body fluids or injured skin	4.1 (3)	9.8 (11)	2.9 (4)	4.7 (5)	36.2 (5)	…	5.4 (14)
**Tasks during which accident occurred**							
Blood sampling	46.6 (34)	8 (9)	30 (41)	0.9 (1)	…	50 (2)	16.8 (44)
Infusion	5.5 (4)	3.5 (4)	9.5 (13)	…	…	…	5 (13)
Surgery	…	11.5 (13)	…	…	50.7 (7)	150 (6)	5 (13)
Nursing, hygiene	5.5 (4)	4.4 (5)	5.9 (8)	3.7 (4)	…	…	4.6 (12)
Injection	4.1 (3)	4.4 (5)	5.1 (7)	…	14.5 (2)	…	3.4 (9)
Other care (eg, hemodialysis, catheter manipulation)	4.1 (3)	2.7 (3)	5.1 (7)	2.8 (3)	…	…	3.8 (10)
Other tasks apart from direct contact with the patient (eg, cleaning objects)	5.5 (4)	0.9 (1)	2.2 (3)	3.7 (4)	…	…	2.7 (7)
Total	46.1 (52)	53.0 (40)	73.8 (79)	8.8 (12)	65.2 (9)	200 (8)	41.3 (108)

a Indicence per 1,000 person years; the number of accidents is specified in brackets, unless otherwise indicated.

Note. Other medical specialties: cardiology, geriatrics, gastroenterology, infectious diseases, internal medicine, nephrology, oncology, pulmonology, rheumatology, urology.

### Bivariate and multivariate analyses on predictors of AEBs among HCWs

Table 2 shows the results of bivariate analyses conducted on variables associated with AEBs among HCWs. These analyses helped identify potential individual-level predictors that might be associated with the occurrence of AEBs in HCWs. Table 3 presents the multivariate model selected using Akaike information criterion (AIC), which shows significant association between the occurrence of AEBs and various individual-level predictors. These predictors included younger age (relative risk [RR], 4.25; 95% confidence interval [CI], 1.20–9.94; *P* = .026), occupation as nurses (RR, 2.43; 95% CI, 1.25–4.52; *P* = .009) or midwives (RR, 2.90; 95% CI, 1.32–4.45; *P* = .012), irregular work schedules (RR, 3.18; 95% CI, 1.83–5.11; *P* < .001), rotating shifts (RR, 3.11; 95% CI, 1.72–4.83; *P* = .001), and lack of support from supervisors (RR, 1.16; 95% CI, 1.06–1.28; *P* = .001). No significant variation over time was observed. Additionally, the use of external nurses was a significant ward-level predictor associated with the occurrence of AEBs (RR, 2.02; 95% CI, 1.19–3.35; *P* = .010).

**Table 2.** General and Organizational Characteristics of HCWs on Occupational Exposure to Blood Using Bivariate Analysis (Logistic Regression)

Characteristic	Participants at Inclusion (N=730)	Overall Incidence Rate of AEBs per 1,000 Person Years	Overall AEBs, (N=71), No. (%)^a^	*P* Value
Time of visit				.50
T0	730	103	25 (35)	
T1 (T0 + 4 mo)	680	66	15 (21)	
T2 (T0 + 8 mo)	612	69	14 (20)	
T3 (T0 + 12 mo)	591	86	17 (24)	
Hospital				.004*
A	159	11.0	6 (8.5)	
B	174	16.8	10 (14)	
C	173	35.8	23 (32)	
D	224	38.4	32 (45)	
**Provider variables**				
Age range, y (SD)				<.001*
>55	66	8.3	2 (2.8)	
46–55	132	10.3	5 (7.0)	
36–45	154	23.0	13 (18)	
26–35	253	27.1	24 (34)	
≤25	125	61.4	27 (38)	
Sex				.30
Male	120	20.5	9 (13)	
Female	610	28.5	62 (87)	
Occupation				<.001*
Nursing assistant	300	10.3	11 (15)	
Nurses	384	35.2	48 (68)	
Physician	35	36.2	5 (7.0)	
Midwife	11	175.0	7 (9.9)	
Occupational status				.018*
Permanent	663	24.8	59 (83)	
Contractual	66	51.3	12 (17)	
ND	1			
**Work variables**				
Supervising responsibility				>.90
No	330	27.7	33 (47)	
Yes	380	27.6	37 (53)	
ND	20		1	
Schedule known in advance				.025*
Sometimes	88	6.8	2 (3.0)	
Always	614	29.0	64 (97)	
ND	28		5	
Involvement in schedule planning				.70
Never	322	23.5	27 (41)	
Always	112	27.4	11 (17)	
Sometimes	268	29.4	28 (42)	
ND	28		5	
48-h weekly rest				.20
No	302	21.5	23 (35)	
Yes	400	30.0	43 (65)	
ND	28		5	
Type of work shift				<.001*
Day only	493	20.9	37 (52)	
Night only	199	27.6	20 (28)	
Rotating shift (night and day)	38	116.7	14 (20)	
Schedule allocation frequency				<.001*
Regular	620	23.1	54 (79)	
Irregular	77	70.7	14 (21)	
ND	33		3	
On-call or overnight shift				.06
No	566	22.3	36 (54)	
Yes	130	34.9	31 (46)	
ND	34		4	
Modified work hours				.20
Sometimes	551	24.7	48 (73)	
Often	144	33.9	18 (27)	
ND	35		5	
Working overtime				.003*
Sometimes	412	18.6	27 (41)	
Often	283	37.9	39 (59)	
ND	35		5	
Meal time				.008*
Regular	103	7.3	3 (4.5)	
Irregular	591	30.5	63 (95)	
Unknown	36		5	
Break cancellation				.004*
Sometimes	177	13.3	11 (17)	
Often	518	33.3	55 (83)	
Unknown	35		5	
Commuting time to work				.004*
>2 h	103	12.7	4 (5.6)	
1–2 h	321	19.6	23 (32)	
<1 h	306	39.1	44 (62)	
Mode of transport				.007*
Personal car	365	17.0	22 (31)	
Public transportation	303	36.6	40 (56)	
Other (walking, bike, motorbike)	62	39.5	9 (13)	
Intention to leave job				.015*
No	410	19.6	27 (39)	
Yes	320	35.0	43 (61)	
ND			1	
**Hospital survey on patient safety culture**				
Item 1: “Hospital management provides a work climate that promotes patient safety.”				.20
Agree	220	24.3	51 (72)	
Neutral	115	34.8	4 (5.6)	
Do not agree	395	40.5	16 (23)	
Item 2: “The actions of hospital management show that patient safety is a top priority.”				.50
Neutral	102	19.6	2 (2.8)	
Agree	230	26.0	55 (77)	
Do not agree	398	35.2	14 (20)	
Item 8: “Hospital management seems interested in patient safety only after an adverse event happens.”				.60
Agree	492	26.5	63 (89)	
Neutral	93	32.3	3 (4.2)	
Do not agree	145	34.5	5 (7.0)	
Item 10: “Hospital units work well together to provide the best care for patients.”				.15
Agree	431	25.1	58 (82)	
Neutral	125	40.0	5 (7.0)	
Do not agree	174	46.0	8 (11)	
**HCW perception (validated scales)**				
Social support from supervisor, Karazek subscale (of 16)				.90
Good support (score ≥13)	241	27.2	20 (0.8)	
Little support (score <13)	489	27.1	51 (2.0)	
Social support from colleagues – Karazek subscale (of 16)				.037*
Good support (score ≥13)	337	34.8	45 (1.7)	
Lack of support (score <13)	328	20.8	26 (1.0)	
Overinvestment at work – Siegrist subscale (of 24)				.003*
Moderate investment (score <17)	477	20.8	37 (1.4)	
High investment (score ≥17)	253	40.8	34 (1.3)	
Stress, PSS-10 scale (of 40)				.30
Moderate stress (score <28)	681	28.2	69 (2.6)	
High stress (score ≥28)	49	12.0	2 (0.1)	
Fatigue, Pichot scale (of 32)				.80
Moderate fatigue (score <23)	531	26.5	48 (0.9)	
High fatigue (score ≥23)	199	28.6	23 (0.9)	
**Ward variables**				
Specialty				.20
Other medical specialties^b^	213	18.5	14 (20)	
Surgery, obstetrics	314	28.4	32 (45)	
ICU	203	34.2	25 (35)	
Use of external personnel, nurses				<.001*
Sometimes	357	15.7	20 (28)	
Often	373	38.1	51 (72)	
Use of external personnel, nursing assistant				.50
Sometimes	446	24.9	40 (56)	
Often	284	30.7	31 (44)	
Use of outsourcing staff, nurses				.21
Sometimes	552	23.4	46 (65)	
Often	178	38.9	25 (35)	
Time schedule				.60
3×8 h	577	26.3	54 (76)	
2×12 h	153	30.2	17 (24)	

Note. AEB, accidental exposure to blood; HCW, healthcare worker; ICU, intensive care unit; ND, No Data Available; SD, standard deviation.

a No. (%) unless otherwise indicated.

b Other medical specialties: cardiology, geriatrics, gastroenterology, infectious diseases, internal medicine, nephrology, oncology, pulmonology, rheumatology, urology.

* Indicates statistical significance.

**Table 3.** Multivariate Analysis of Accidental Exposure to Blood Using a Stepwise Linear Mixed-Effects Model Selection Based on Akaike Information Criterion

Variable	Bivariate Analysis			Multivariate Analysis		
	RR	95% CI^a^	*P* Value	RR	95% CI^a^	*P* Value
**Fixed effects**						
Time of visit						
t0	…	…		…	…	
t1	0.63	0.33–1.20	.17	0.75	0.39–1.47	.4
t2	0.65	0.34–1.23	.18	0.75	0.38–1.51	.4
t3	0.81	0.45–1.49	.51	0.80	0.41–1.55	.5
**Provider variables**						
Occupation						
Nursing assistant	…	…		…	…	
Nurse	3.17	1.74–5.55	<.001	2.43	1.25–4.52	.009
Physician	3.38	1.27–8.02	.016	2.82	0.61–10.1	.2
Midwife	4.52	3.27–5.23	<.001	2.90	1.32–4.45	.012
Age range, y						
>55	…	…		…	…	
46–55	1.46	0.29–7.21	.64	1.40	0.27–7.01	.7
36–45	3.24	0.77–11.5	.11	2.69	0.61–10.2	.2
26–35	3.68	0.94–11.8	.06	2.46	0.59–8.83	.2
≤25	6.06	2.00–11.7	.003	4.25	1.20–9.94	.026
**Work variables**						
Work schedule consistency						
Regular	…	…		…	…	
Irregular	2.62	1.55–4.17	<.001	3.18	1.83–5.11	<.001
Type of work shift						
Daily	…	…		…	…	
Nightly	1.31	0.77–2.20	.32	1.67	0.93–2.93	.085
Rotating shift (night and day)	3.58	2.27–5.04	<.001	3.11	1.72–4.83	<.001
Lack of support from supervisor^a^	1.13	1.04–1.23	.003	1.16	1.06–1.28	.001
**Ward-level variables**						
Use of external personnel (nurses)						
Sometimes	…	…		…	…	
Often	2.28	1.39–3.65	.001	2.02	1.19–3.35	.010

Note. RR, relative risk; CI, confidence interval.

a Refers to the perceived support from supervisors as assessed by the Karazek questionnaire. This subscale measure the extent to which HCWs perceive their supervisors to be unsupportive or indifferent to their needs and concerns. A higher score indicates a greater perceived lack of support from supervisors, which has been identified as a potential risk factor for occupational blood exposure.

## Discussion

The main findings of this longitudinal study highlight the importance of considering both individual and organizational factors when addressing AEBs among HCWs. We identified several significant factors, including occupation, age, work schedule consistency, rotating shifts, social support from supervisors, and the frequent use of external nurses. However, there was no evidence of relationship between stress and fatigue and the occurrence of AEBs.

The study revealed that physicians, nurses, and midwives, who have more frequent and direct contact with patients, are more likely to be exposed to AEBs.^19^ The increased risk among these occupations could be due to the nature of their work, involving invasive procedures, handling of sharp instruments, and frequent patient interactions.^5^ Additionally, younger HCWs may be at higher risk due to their lack of experience and knowledge of infection control and safety procedures.^20^ Targeted training and education to especially those who are relatively inexperienced is paramount; education has been shown to be effective in reducing AEBs.^21^

Inconsistent work schedules and rotating shifts can increase the risk of AEBs. Our findings suggest that healthcare facilities should consider the impact of work-shift changes and schedule consistency on the health and patient safety.^22^ It is essential to allow HCWs sufficient time to rest and recover between shifts as well as appropriate support to cope with schedule changes. Extended work hours and insufficient rest periods are known to increase AEB risk.^23^ Additionally, occupational injuries can result from consecutive and cumulative shifts.^24^ Prolonged work hours can also lead to sleep disruption, negatively affecting HCW performance.^25^ Thus, healthcare facilities should consider implementing strategies, such as shorter work hours, flexible scheduling, and regular breaks during shifts, to mitigate AEB risks associated with work schedules and shift rotation.

Insufficient support from supervisors can lead to increased stress among HCWs, negatively influencing their health, morale, and productivity.^26^ Healthcare facilities should promote social support and safety climate among their staff^8^ through regular meetings with supervisors to discuss work-related challenges, constructive feedback, and a positive work environment.^27^ Factors such as work environment, teamwork, burnout, and personal circumstances can influence the intent of European nurses to leave their job.^28^ Addressing these factors is essential for staff retention. By fostering a supportive culture and adequate nurse staffing, healthcare organizations can decrease AEB risk and improve overall staff safety and quality of care.^29^ Support from supervisors is essential in reducing AEB risk because it promotes a positive safety culture and HCW adherence to safety protocols.^30^

Using external staff in healthcare facilities can result in various issues, including increased risk of infection, accidents, and challenging work conditions.^31^ We hypothesize that this utilization of external staff may serve as a marker for unit staffing instability and culture. Staff operating in multiple healthcare facilities may act as a vector for spreading infections between these locations.^32^ Moreover, HCWs may be more susceptible to accidents given their unfamiliarity with equipment or facility layout, increasing the risk of falls, needle-stick injuries, and other mishaps.^33^ The association between care left undone and temporary nursing staff ratios in acute-care settings underscores the need to address staffing for patient safety.^34^ These staff members might encounter work-related challenges such as job insecurity, dissatisfaction, and burnout. Such challenges arise due to disparities in training and support compared to permanent staff.

Despite these findings, generalizing our results to other hospitals or countries may be limited due to potential variations in organizational practices, prevention policies, cultural contexts, regulations, and available resources in different healthcare settings.^35^ Moreover, healthcare systems and staffing models can differ significantly between regions, potentially influencing the dynamics of AEB risks.

Underreporting of AEBs is a crucial concern with significant implications.^36^ The main causes of underreporting include fear of negative consequences such as stigmatization, legal liability, or disciplinary actions, as well as insufficient awareness, knowledge, and training on reporting procedures.^37^ Additionally, time constraints and complex reporting systems contribute to staff reluctance to report AEBs.^30^ The underreporting of AEBs prevents efforts to improve healthcare worker safety and hinders the development of effective interventions to minimize the risk of infection transmission.^38^ It also perpetuates a culture of secrecy, rather than fostering an open and transparent environment in which learning from incidents is encouraged.

The study has several strengths that enhance its validity and reliability. It was a multicenter study encompassing diverse HCWs and specialties across 4 hospitals. The longitudinal design provided a comprehensive view of the occupational blood exposure effects over time, allowing for trend analyses. Moreover, a combination of diverse metrics, including individual and organizational factors, as well as 2 levels of data (ward level and HCW level), enriched the understanding of factors influencing HCW health outcomes. We used validated scales for measuring stress, fatigue, overinvestment, social support, and human resources data (absenteeism, turnover) to facilitate the identification of contributing factors. Finally, the study was conducted in 2019, before the COVID-19 pandemic, which significantly altered healthcare organization worldwide.^39^ Hence, our findings over the 12-month period were likely unaffected by the effects of the pandemic, an essential consideration when interpreting the results.

This study had several limitations. Data collection regarding physicians’ work characteristics, particularly work hours and shifts, was imprecise. Self-report bias and accident underreporting might have influenced our findings, with potential recall or social desirability bias. Accident underreporting could result from HCW hesitation to report incidents of exposure due to fear of retaliation or reluctance to admit mistakes. This issue may hinder supervisors from providing necessary support and resources, making it difficult to track and prevent future incidents of exposure. We were not able to measure the underreporting of AEBs. In addition, our findings did not establish a direct link between stress, fatigue, and AEBs, possibly due to the presence of confounding factors. Measurements were taken at a single time point, possibly inducing measurement bias. Although AEB data were collected over a longer 4-month period, conclusive evidence of a relationship between stress and fatigue and infectious risks did not emerge.

The study findings have important implications for healthcare organizations, clinicians, and future research in HCW safety. By understanding factors associated with AEBs, facilities can develop targeted interventions addressing risks related to occupation, age, work schedule, shift rotation, and supervisor support. These efforts could include additional training, safer work schedules, and promoting a supportive organizational culture. Healthcare facilities should consider the risks of outsourcing staff and should ensure that HCWs are trained and familiar with infection control protocols to minimize AEBs and other adverse outcomes. HCWs should be encouraged to accurately report AEBs in a supportive environment that minimizes underreporting, with anonymous systems or educational programs emphasizing the importance of reporting for safety improvement.

Future research should develop robust models integrating clinical and organizational factors to better understand the relationships between stress and fatigue and the occurrence of AEBs. Utilizing alternative analytical approaches, such as directed acyclic graphs (DAGs),^40^ could reveal previously unidentified relationships, guiding the development of more effective prevention strategies and enhancing HCW safety and patient care.
