# Identifying individual and organizational predictors of accidental exposure to blood (AEB) among hospital healthcare workers: A longitudinal study – ERRATUM

**DOI:** 10.1017/ice.2025.10362

**Published:** 2026-01

**Authors:** René Sosata Bun, Karim Aït Bouziad, Oumou Salama Daouda, Katiuska Miliani, Anastasia Eworo, Florence Espinasse, Delphine Seytre, Anne Casetta, Simone Nérome, Laura Temime, Mounia N. Hocine, Pascal Astagneau

In this article,^1^ the column “Other medical specialties” was omitted from Table 1 due to an error that occurred during typesetting. The correct table appears below. The publisher apologizes for the error.

**Table 1.** Incidence rate of accidental exposure to blood per 1000 person-years by medical specialty and by occupation (n = 108)

Characteristics of occupational	By medical specialty			By occupation				
exposures to blood	Intensive Care Unit (n = 203)	Surgery obstetrics (n = 314)	Other medical specialties* (n = 213)	Nurses (n = 384)	Nursing Assistants (n = 300)	Physicians (n = 35)	Midwives (n = 11)	Total
**Number of visits**	1128	755	730	1070	1365	138	40	2613
**Nature of injury**								
Splash	38.4 (28)	20.4 (23)	10.6 (8)	35.9 (49)	4.7 (5)	36.2 (5)	–	22.6 (59)
Needlestick injury	30.1 (22)	12.4 (14)	10.6 (8)	20.5 (28)	4.7 (5)	29 (4)	175 (7)	16.8 (44)
Cut by other sharp object	2.7 (2)	2.7 (3)	–	1.5 (2)	1.9 (2)	–	25 (1)	1.9 (5)
**Mechanism of accident occurrence**								
By handling a mounted needle	35.6 (26)	22.2 (25)	14.6 (11)	35.9 (49)	1.9 (2)	29 (4)	175 (7)	23.7 (62)
By handling soiled instruments	21.9 (16)	0.9 (1)	1.3 (1)	10.3 (14)	3.7 (4)	–	–	6.9 (18)
By handling a mounted or unmounted syringe	4.1 (3)	1.8 (2)	1.3 (1)	4.4 (6)	–	–	–	2.3 (6)
By handling samples	5.5 (4)	0.9 (1)	–	2.9 (4)	–	–	25 (1)	1.9 (5)
By intervening on a device	–	–	4 (3)	1.5 (2)	0.9 (1)	–	–	1.1 (3)
Other mechanisms at the origin of contact with body fluids or injured skin	4.1 (3)	9.8 (11)	–	2.9 (4)	4.7 (5)	36.2 (5)	–	5.4 (14)
**Tasks during which accident occurred**								
Blood sampling	46.6 (34)	8 (9)	1.3 (1)	30 (41)	0.9 (1)	–	50 (2)	16.8 (44)
Infusion	5.5 (4)	3.5 (4)	6.6 (5)	9.5 (13)	–	–	–	5 (13)
Surgery	–	11.5 (13)	–	–	–	50.7 (7)	150 (6)	5 (13)
Nursing, hygiene	5.5 (4)	4.4 (5)	4 (3)	5.9 (8)	3.7 (4)	–	–	4.6 (12)
Injection	4.1 (3)	4.4 (5)	1.3 (1)	5.1 (7)	–	14.5 (2)	–	3.4 (9)
Other care (eg hemodialysis, catheter manipulation)	4.1 (3)	2.7 (3)	5.3 (4)	5.1 (7)	2.8 (3)	–	–	3.8 (10)
Other tasks apart from direct contact with the patient (eg cleaning objects)	5.5 (4)	0.9 (1)	2.6 (2)	2.2 (3)	3.7 (4)	–	–	2.7 (7)
**Total**	**46.1(52)**	**53.0(40)**	**21.9(16)**	**73.8(79)**	**8.8(12)**	**65.2(9)**	**200(8)**	**41.3(108)**

*Other medical specialties: cardiology, geriatrics, gastroenterology, infectious diseases, internal medicine, nephrology, oncology, pulmonology, rheumatology, urology. Indicence per 1000 person-years, the number of accident (n) is specified under brackets.

## References

[1] Bun RS , Aït Bouziad K , Daouda OS , et al. Identifying individual and organizational predictors of accidental exposure to blood (AEB) among hospital healthcare workers: A longitudinal study. Infect Control Hosp Epidemiol 2024;45(4):491–500. doi: 10.1017/ice.2023.248 38086622 PMC11007361

